# Full-Range Optical Imaging of Planar Collagen Fiber Orientation Using Polarized Light Microscopy

**DOI:** 10.1155/2021/6879765

**Published:** 2021-11-28

**Authors:** Michaela Turčanová, Martin Hrtoň, Petr Dvořák, Kamil Novák, Markéta Hermanová, Zdeněk Bednařík, Stanislav Polzer, Jiří Burša

**Affiliations:** ^1^Brno University of Technology, Faculty of Mechanical Engineering, Institute of Solid Mechanics, Mechatronics and Biomechanics, Technická 2896/2, Brno 616 69, Czech Republic; ^2^Brno University of Technology, Faculty of Mechanical Engineering, Institute of Physical Engineering, Technická 2896/2, Brno 616 69, Czech Republic; ^3^1st Department of Pathology, St. Anne's University Hospital Brno and Faculty of Medicine, Masaryk University, Pekařská 664/53, 656 91 Brno, Czech Republic; ^4^Department of Anatomy, Faculty of Medicine, Masaryk University, Kamenice 126/3, Brno, 625 00, Czech Republic; ^5^Technical University Ostrava, Faculty of Mechanical Engineering, Department of Applied Mechanics, 17 Listopadu 15, Ostrava 708 33, Czech Republic

## Abstract

A novel method for semiautomated assessment of directions of collagen fibers in soft tissues using histological image analysis is presented. It is based on multiple rotated images obtained via polarized light microscopy without any additional components, i.e., with just two polarizers being either perpendicular or nonperpendicular (rotated). This arrangement breaks the limitation of 90° periodicity of polarized light intensity and evaluates the in-plane fiber orientation over the whole 180° range accurately and quickly. After having verified the method, we used histological specimens of porcine Achilles tendon and aorta to validate the proposed algorithm and to lower the number of rotated images needed for evaluation. Our algorithm is capable to analyze 5·10^5^ pixels in one micrograph in a few seconds and is thus a powerful and cheap tool promising a broad application in detection of collagen fiber distribution in soft tissues.

## 1. Introduction

Collagen fibers and their structure are responsible for the distinctive mechanical response of many soft biological tissues such as skin, tendons, cartilages, myocardium, or arteries [1]. Similarly, the observed differences in mechanical behavior (flexibility and strength) between healthy and pathological arteries (e.g., abdominal aortic aneurysm) are largely related to changes in collagen organization [2–6]. Consequently, knowledge of collagen structure in soft tissues is crucial for a deeper understanding of their mechanical behavior and for their structure-based constitutive description [7] which is decisive for clinical applications of computational models. For instance, computational modeling offers the best assessment of the rupture risk of aortic aneurysm or atherosclerotic plaque [4, 8–10] and promises exploitation in timing of surgeries.

There are several approaches how to visualize collagen fibers in soft tissues but each has some limitations. For example, the small-angle X-ray scattering (SAXS) [11] suffers from disadvantages such as degradation of organic fibers, complexity of optical elements, and need for safety precautions to prevent inadvertent irradiation. Another frequently used method, small-angle light scattering (SALS) [12], is precise but also highly time-consuming. Fluorescence microscopy-based methods, such as confocal laser scanning microscopy (CLSM) [13, 14] or multiphoton microscopy (MM) [15], are precise and fast but require expensive microscopic equipment and mostly a complex sample staining with combination of adhesion protein (CNA35) and a special fluorescent dye (OG488) [13, 14, 16]. Noncentrosymmetric crystalline triple helix structure of collagen allows to emit second harmonic generation (SHG) light used in multiphoton or confocal microscopy [17]. Tanaka et al. used polarization-resolved SHG microscopy to show the orientation of collagen in human facial skin in vivo [18]. This method has proven to be particularly suitable for the observation and analysis of collagen orientation in relatively simple tissues, e.g., in the skin or tendon but is not very suitable for more complex tissues (vessel wall) [18]. Although quantitative polarized light microscopy (QPLM) [19–21] can be more effective here, it requires a sophisticated and rather expensive equipment: a modified confocal microscope with quarter-wave plate and rotating analyzer. While the above studies demonstrated the ability of QPLM to determine the orientation of collagen, they did not address the dispersion of fibers and its subsequent use in constitutive models. For this purpose, fast Fourier transform is often applied [22–24] despite of its drawback increasing its time demands: it evaluates the dominant fiber directions correctly [22] but requires calibration by ground truth data to correctly capture the dispersion of fiber directions [23].

In contrast, classical polarized light microscopy (PLM) is a simple and widely used optical method preferred in analyses of collagen structure because it allows evaluation over a large sample area and does not require any expensive equipment or complex sample preparation [4, 22, 25–27]. It is based on two mutually perpendicular (crossed) polarizers that transmit only a portion of the polarized light scattered by a birefringent specimen. Nevertheless, this method has two limitations: first, in case of manual evaluation, it is very slow and sensitive to operator's experience and concentration when following the chosen fiber till its extinction during specimen rotation. Its inaccuracy and operator's dependence were documented by Novak et al. [28]. Consequently, a typical number of manually evaluated points (volume elements) per image is of the order of tens [4, 22, 27] which is not sufficient to extract rigorous information regarding dispersion of collagen fibers. Second, two mutually perpendicular structures cannot be distinguished from each other due to its intrinsic 90° uncertainty originating from the periodic nature of the pixel angular intensity profile (i.e., dependence of pixel intensity on the sample rotation).

In this paper, we present a new method for semiautomatic evaluation of the orientation and dispersion of collagen fibers using PLM within all the 180° range without any additional components. Although this limitation has already been overcome by addition of a universal compensator (two variable retarders) in front of the sample [29–33], or a quarter-wave plate behind it [34, 35] in the light path of the polarizing microscope, the presented algorithm is applicable with any polarized light microscope even if this additional equipment cannot be used. We have found another way how to overcome the angular limitation given by the periodicity of light intensity at crossed filters and proposed an automatic algorithm that is capable to evaluate the orientation in each pixel of the micrograph. The obtained histograms, transformed into mean angles of fibers and parameters of their concentration (dispersion), serve as inputs to structural constitutive models of the tissues in computational modeling, e.g., of healthy and pathological states of blood vessels.

## 2. Materials and Methods

In manual PLM with crossed polarizers, the specimen orientation with zero intensity (extinction) is seeked for, while in automated approach, the intensity values measured under different specimen rotations are approximated with a sine function with *π*/2 periodicity to find the angle of minimal intensity [26]. To overcome this intrinsic limitation, we need to double this periodicity.

### 2.1. Procedure Description and the Proposed Automated Algorithm

**Figure 1:**
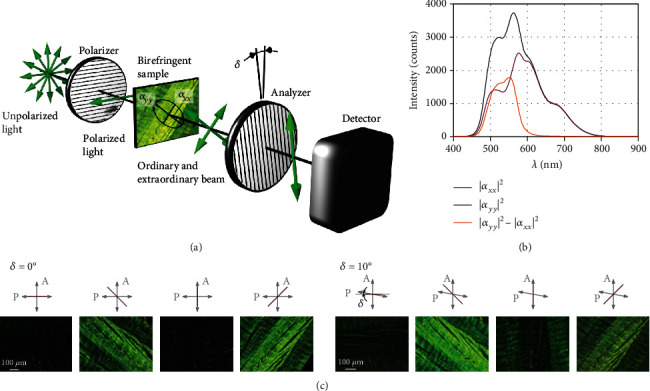
(a) Experimental scheme of a polarized light microscope with unidirectional fibers. (b) Spectral dependence of the transmission matrix components *α*_*xx*_, *α*_*yy*_, and their difference. Anisotropy of collagen fibers is most pronounced at wavelengths between 550 and 570 *μ*m, as illustrated by the difference between |*α*_*xx*_|^2^ and |*α*_*yy*_|^2^, which represent light intensities of ordinary and extraordinary beams transmitted through the specimen with parallel setting of polarizers. (c) Images of a sample of porcine Achilles tendon with polarizers being either perpendicular (four images in the left) or rotated by angle *δ* (four images in the right).

The standard PLM setting with cross-polarized filters, i.e., with perpendicular orientation of polarizer 1 and polarizer 2 (called analyzer below) is shown in Figure 1(a). Consequently, no light can pass through both polarizers, unless its polarization state is changed by a birefringent (optically anisotropic) material located in the light trajectory between both polarizers. The birefringent material rotates the plane of polarized light towards its major axis of anisotropy so that this plane is no longer perpendicular to the analyzer, and some portion of the light can pass through. The intensity of the transmitted light depends on its polarization and is maximal (or minimal) for the light polarized in two mutually perpendicular directions [36]. Zero intensity (fiber extinction) occurs when the fiber is oriented in parallel with either the polarizer or the analyzer, and the intensity of the passing light is maximal for the fiber angle of 45° with respect to both polarizers.

Another important feature of collagen is its diattenuation, a total anisotropic attenuation of light caused by absorption and scattering. To choose most convenient parameters of the setting, the diattenuation was measured for different light wavelengths (see Figure 1(b)) using a fiber optic spectrometer StellarNet BW-VIS2 (with 600 *μ*m aperture of optical fiber); the signal originated from a small circular area (30 *μ*m in diameter) in the center of a specimen of collagen tissue. The proposed method exploits combination of these two effects to switch the angular periodicity of the transmitted light from 90° to 180° for nonperpendicular polarizers. This is illustrated with images of porcine tendon in Figure 1(c) where differences of intensity between the neighboring maxima are visible for the rotated analyzer (*δ* = 10°).

In contrast to manual measurements, the automated algorithms record the polarized light intensities under different rotations of the specimen and subsequently fit the measured data with a theoretical intensity curve. The proposed algorithm builds on our approach reported in [28] which exploited a well-known phase-correlation and 90° cosine-like periodicity of the polarized light intensity under 2D (in-plane) rotation of the specimen. Within this approach, three rotated images were needed to fit the position of the purely cosine-like normalized intensity curve which, however, cannot suffice here due to nonsinusoidal character of the periodic function.

**Figure 2:**
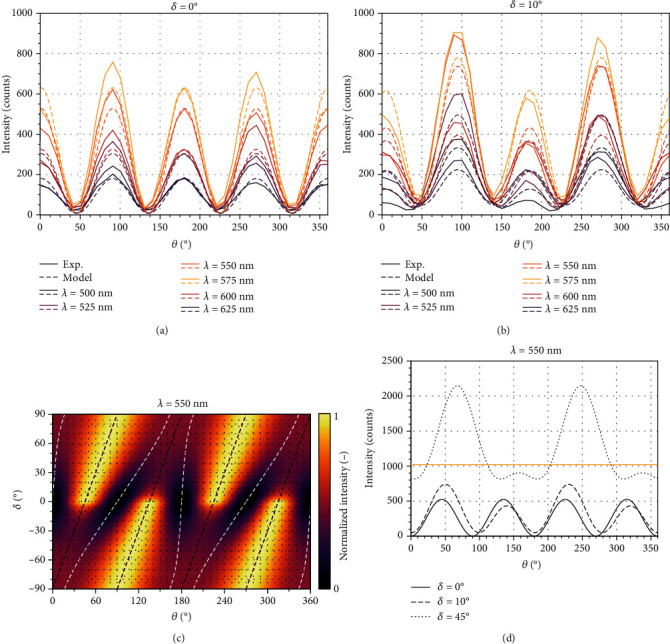
(a) and (b) Dependences of the intensity of transmitted polarized light on the fiber angle *θ* for six different wavelengths *λ* and two different analyzer rotation angles *δ* measured using a fiber spectrometer; our model adequately reproduces the experimental results. (c) Intensities of green light with wavelength *λ* = 550  nm for different analyzer angles *δ* and sample rotation angles *θ* (the fiber angle *p* is implicitly set to 0°). Black and white dotted lines represent maxima and minima of the intensity calculated for a certain value of *δ*, respectively. (d) Horizontal sections through the graph (c), representing functions of sample rotation angle *θ* for three different analyzer angles *δ*. Solid line (*δ* = 0°) shows 90° periodicity while the other angles show 180° periodicity with each second maximum being highly suppressed. Extreme differences in intensities between *δ* = 0° and *δ* = 45° disable practical application of this combination of angles, as the intensity curve for *δ* = 45° is above the pixel saturation value for most *θ* angles (shown by the yellow line in the graph). However, when using another camera with higher saturation level or different setting, another *δ* value can be used and introduced easily into the algorithm.

Before measuring, we calibrated both polarizers and set the coaxiality of the axis of specimen rotation with the optical axis. In the first step, we recorded a set of 18 images rotated per 10° with cross-polarized setting (i.e., *δ* = 0°, see Figure 2(a)). Then, the algorithm used phase correlation procedure [37] to rotate and shift all the images to aligned positions; we note this step would not be needed if the polarizers were rotated instead of the specimen. Then, it evaluates the light intensities in corresponding pixels of all the rotated images, and their values are fitted with a chosen cosine-like function in the following form:

(1) $$\begin{array}{c} I\left(\theta \right)=\frac{1}{2}-\frac{1}{2}\cos\left[4\left(\theta -p\right)\right], \end{array}$$

where *θ* denotes the rotation angle of the sample with respect to the polarizer, and *p* represents the angular (phase) shift of this function determining the fiber orientation in the investigated pixel. This parameter is fitted to obtain the best correlation with the measured values which is in turn achieved by maximizing Pearson's correlation coefficient (see [28] for more details).

The minima in the cosine-like function represent the angles, at which the fiber in the investigated pixel is oriented along the polarization axis of either the polarizer or the analyzer. As one cannot distinguish between these two perpendicular orientations, this ambiguity is resolved by setting *δ* ≠ 0°, i.e., by employing rotated polarizers (the polarizer axes are nonperpendicular to each other). A full mathematical derivation of the intensity profile in this setting can be found in Supplementary material (available here); here, we present only the final formula:

(2) $$\begin{array}{c} I\left(\theta \right)=\frac{1}{8}{\left|\alpha_{xx}-\alpha_{yy}\right|}^{2}\left[1-\cos\left(4\left(\theta -p\right)\right)\cos2\delta -\sin\left(4\left(\theta -p\right)\right)\sin2\delta \right]+\frac{1}{4}\left({\left|\alpha_{xx}\right|}^{2}-{\left|\alpha_{yy}\right|}^{2}\right)\left[\cos\left(2\left(\theta -p\right)\right)\left(1-\cos2\delta \right)-\sin\left(2\left(\theta -p\right)\right)\sin2\delta \right]+\frac{1}{8}{\left|\alpha_{xx}+\alpha_{yy}\right|}^{2}\left(1-\cos2\delta \right), \end{array}$$

where *α*_*ii*_ represents the transmission amplitude of light polarized along the principal axis *i* (see Figure 1(b)).

The first term (~ |*α*_*xx*_ − *α*_*yy*_|^2^) describes the original intensity profile with 90° periodicity, while the second term (~|*α*_*xx*_|^2^ − |*α*_*yy*_|^2^) possesses periodicity twice as large and overcomes thus the limitation inherent to the cross-polarized configuration. Note that apart from the mutual rotation of the polarizers, the sample is also required to exhibit significant diattenuation (|*α*_*xx*_|^2^ − |*α*_*yy*_|^2^ ≠ 0), a prerequisite satisfied in collagen fibers. The procedure for determining the terms containing various mixtures of transmission amplitudes *α*_*xx*_ and *α*_*yy*_ is briefly outlined at the end of the Supplementary material.

Therefore, the second step of our algorithm consists of recording another set of 18 images with rotated polarizers (*δ* = 10°), which are then used to select the true orientation of fibers in our specimen from the two possible options found in the previous step as described below.

Examples of the intensity curves, both measured and calculated (using eq. (2)), are presented in Figures 2(a) and 2(b). Slight deviations of the measured curves from the periodical theoretical dependences are caused likely by the fact that we rotate the sample and, despite our best efforts devoted to calibration, the spot from which these particular spectra were taken does not coincide exactly with the rotation axis of our sample stage. However, as these discrepancies concern only amplitude and not the phase of the *π*/2 periodic curves for the crossed polarizers (*δ* = 0°), they have no impact on the evaluated fiber angle. In contrast, a pronounced difference occurs between even and odd local maxima for *δ* = 10°, enabling us to distinguish between the two mutually perpendicular orientations. This difference between the neighboring local maxima depends on the wavelength; on the basis of measurements and simulations, the wavelength of 550 nm (green color) was chosen as the best candidate, since it provides the largest difference of more than 50% between |*α*_*xx*_|^2^ and |*α*_*yy*_|^2^, as shown in Figure 1(b). To choose optimal *δ* for this wavelength, we calculated the dependence of light intensity on sample rotation angle *θ* and analyzer angle *δ* (for its full range between -90° and +90°) using the measured transmittance data from Figure 1(b); the resulting intensity map is plotted in Figure 2(c).

This map shows that for *δ* ≳ 5°, each second maximum is significantly reduced and thus the periodicity changes to 180°. Cuts throughout the intensity map in Figure 2(c) for analyzer angles *δ* of 0°, 10°, and 45° show this phenomenon, making the minima more flat and hardly detectable (see Figure 2(d)). Therefore, the algorithm exploits the larger (nonreduced) maximum instead. As soon as the larger maximum is found, the angle of minimum intensity corresponds to the position of the minimum to the left (right) of this maximum for positive (negative) analyzer angle *δ*, respectively (see Figure 2(c)). We have chosen *δ* = 10° because a higher deviation may cause overexposure of the image (see Figure 2(d)) where a high increase in light intensity is shown for deviation *δ* = 45°. Moreover, the shift between maxima of the *π*/2-periodic and *π*-periodic intensity functions increases with angle *δ* and reaches values close to 90° for *δ* = 45° (see Figures 2(c) and 2(d)); this could make the switching between left and right minima sensitive to additional errors. Note that one could, in principle, extract the fiber orientation solely from the set of images with rotated polarizers, i.e., from the dashed or dotted curve in Figure 2(d). However, the inaccuracies in the evaluation of the relatively flat minima in the nonsinusoidal function, together with inaccuracies in the measured diattenuation parameters of the sample, render this approach rather unreliable. This apparent susceptibility to errors was the main reason standing behind our decision to adopt the more robust two-step procedure described above.

Note that for specimens possessing only a weak anisotropy, the background birefringence can play an important role and one should compensate for it in the data analysis using, for example, a procedure analogous to that outlined in [29].

### 2.2. Preparation of Samples

The proposed method was validated using porcine Achilles tendon and porcine aortic wall. Specimens were harvested in a local slaughterhouse from 10 months old pigs weighing 105-120 kg. The preparation of histological sections was done in St. Anne's University Hospital in Brno.

Achilles tendon consists of unidirectionally oriented collagen fibers being prone to undulation if an unloaded tendon is used to harvest the specimen. Cuboid specimens of approx. 20 × 8 × 8 mm were cut off and fixed in 10% formaldehyde solution (at room temperature for 24 hours) to prevent autolytic changes and consequent degradation of the tissue. After that, the samples were dehydrated and embedded in paraffin. Microtome was used to cut 5 *μ*m thick slices parallel to the preferred (longitudinal) direction of collagen fibers. Finally, every slice was stained with a special dye Picro Sirius Red (PSR) (0.1%) to intensify the birefringence of collagen [38].

Aortic specimens with dimensions 18 × 18 mm were taken from the anterior wall of straight part of thoracic aorta. They were flattened with four pins on a plywood, and the slice cut in circumferential-axial plane was treated in the same manner.

### 2.3. Experimental Setup

All the histological slices were scanned with an upright microscope (Padim, Drexx s.r.o., CZ) in a transmission configuration equipped with a digital camera (Bresser microcam 5 megapixel, Bresser GmbH, Germany) and standard 2D rotary stage (Padim, Drexx s.r.o., CZ). All the images were recorded under identical illumination conditions and magnification (10× objective, numerical aperture 0.17; 10× ocular, exposure time 500 ms); the trimmed images had the size of 960 × 960 pixels (pixel size 0.73 *μ*m). A halogen lamp with a power of 100 W was used as a white light source.

## 3. Results

### 3.1. Verification of the Algorithm

**Figure 3:**
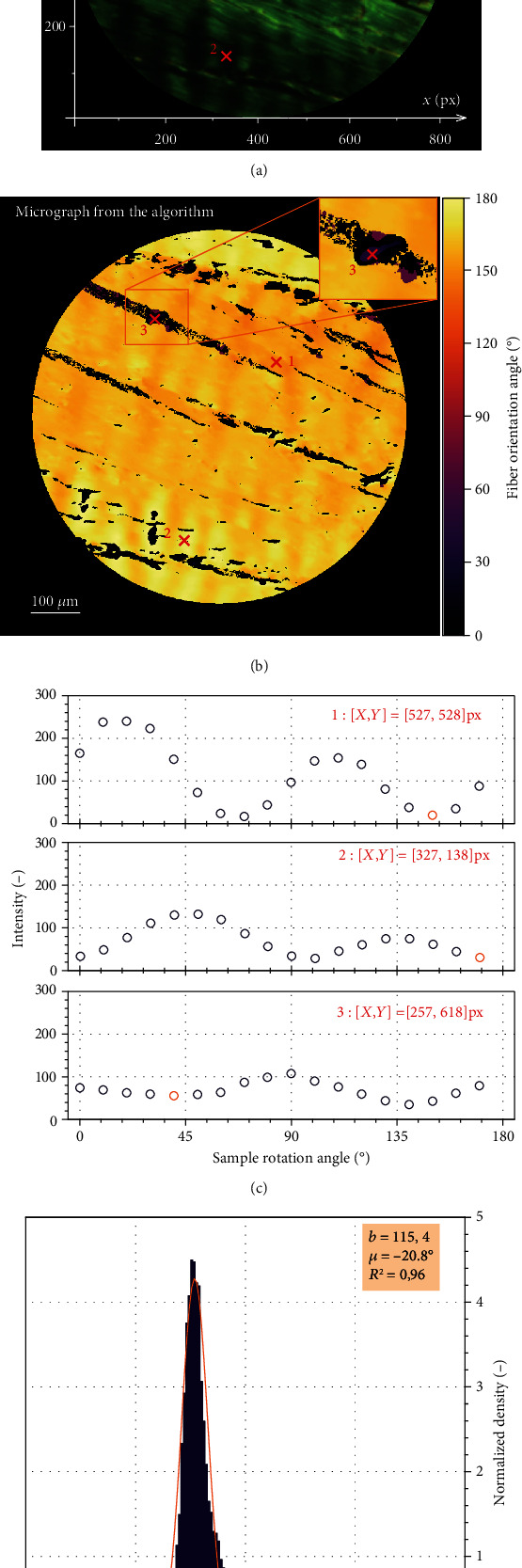
Verification of the algorithm at three different locations of porcine Achilles tendon sample. Figure (a) shows a micrograph (the part with pixel 3 is zoomed in upper right corner) with a setting for green color and *δ* = 0°. The micrograph with directions evaluated by the proposed algorithm is shown in color scale in (b). Figure (c) shows the light intensity dependence on the rotation angle in the selected three pixels (with specification of their coordinates in Fig. (a)) of the sample image recorded with rotated polarizers. The minimum to the left of the main maximum indicates the angle of the fiber (orange circle) which is detected accurately from the *π*/2 periodic intensity function from the first set. In this way, also the pixel 3 is evaluated correctly and its highly different orientation which is visible in the histogram. The histogram obtained for all pixels is interpolated in figure (d) with the von Mises distribution function, and its parameters are listed in the legend. Here, the angles are rescaled between -90° and +90° (−20° = 160°) to avoid cutting of the distribution at 0° and to keep common conventions for arteries.

As the functionality of the original algorithm with *π*/2 periodicity was already validated on artificial images in [28], we verified only the proposed extension to 180° periodicity, i.e., how the algorithm distinguishes between two mutually perpendicular directions. To this end, a sample of porcine Achilles tendon was chosen because the orientation of its unidirectional slightly wavy fibers can be easily measured manually (see Figure 3(a)). The light intensity curves were evaluated with both perpendicular and rotated polarizers for three selected pixels where the fiber in pixel 3 is nearly perpendicular to the fiber orientation dominating the majority of the sample. First, the orientation of the fiber in each pixel (measured as the deviation from the horizontal direction) is determined from the *π*/2-periodic intensity function (recorded with perpendicular polarizers). Then, the main maxima which grow in magnitude when recorded with rotated polarizers (for *δ* = 10°) are recognizable in the waveforms in Figure 3(c), and the fiber angle is determined as the angle of the minimum located just to the left of the main (larger) maximum in the corresponding *π*-periodic intensity function in this figure. Thus, the second set of images measured with rotated polarizers serves only as a logic gate that allows us to choose between the two mutually perpendicular orientations of the fibers determined in the first step. Finally, to obtain structural parameters needed for constitutive models, the resulting histogram (Figure 3(d)) is interpolated with the following von Mises distribution function [39]:

(3) $$\begin{array}{c} \rho \left(\varphi \right)=\frac{\exp\left(b∙\cos\left(\varphi -\mu \right)\right)}{2∙\pi ∙I_{0}\left(b\right)}, \end{array}$$

where *b* is the concentration parameter, *φ* is the pixel-wise evaluated fiber angle, *μ* is the mean direction of the distribution, and *I*_0_(*b*) is Bessel function. The quality of this approximation is evaluated by the coefficient of determination *R*^2^. Coefficient of determination *R*^2^ indicates the proportionate amount of variation in the response variable *y* explained by the independent variables *X* in the linear regression model. The larger *R*^2^, the more variability is explained by the linear regression model [40]. This approximation and its parameters are shown with the histograms in all relevant figures.

### 3.2. Validation of the Algorithm

**Figure 4:**
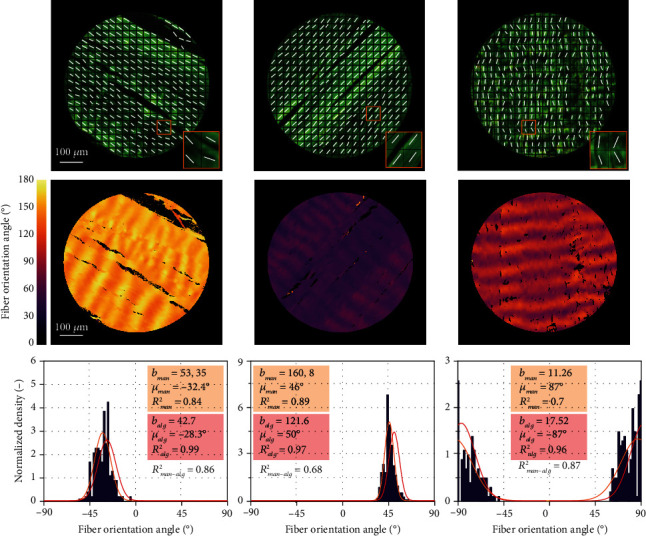
Validation of the algorithm with porcine Achilles tendon using manual measurement of fiber angles in the marked areas. The micrographs in the upper row show c. 300 square-shaped regions with the fiber angles determined by the operator. The images in the middle row show (in color scale) the fiber orientation evaluated by the algorithm. Histograms from the manual measurements are displayed in the bottom row, completed in the legends with parameters of the von Mises distributions fitted to the manual measurements (with subscripts man) and to the algorithm results (with subscripts alg).

Figure 4 shows the validation results, i.e., comparison between the automated algorithm and manual measurements. The results of the manual measurement, with some 300 square-shaped evaluated areas (each of approx. 39 × 39 pixels), are presented in histograms. Deviations between the parameters of the manual and automated evaluation (between 4° and 6° for the mean angle) can be attributed to the (by orders) smaller numbers of areas evaluated manually. This is indicated by their lower *R*^2^_man_ values (coefficient of determination of the fit to manual measurements) while for the automated algorithm the approximation of histograms is perfect (*R*^2^_alg_ > 0.96). The manual histograms suffer evidently from low numbers of measurements and consequently from an impact of noise, as confirmed by a better accordance of two less accurate fitting curves of manual data with the fit of the algorithm data (represented by *R*^2^_man−alg_ in Figure 4) than with the manual measurements themselves (*R*^2^_man_).

### 3.3. Number of Images Needed for Detection of Orientation of Collagen Fibers

**Figure 5:**
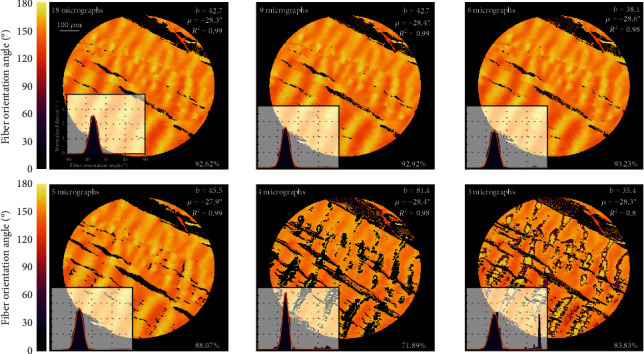
Comparison of the resulting histograms and parameters of their von Mises approximations obtained with different numbers of input micrographs of the porcine Achilles tendon. The sample was intentionally oriented under the angle of approx. 152° = −28°.

The presented procedure exploits two sets of 18 images with rotation step of 10°. To reduce the time-consumption of this approach, we tested what is the minimum number of images necessary to detect the correct orientation of collagen fibers without a significant loss of information or accuracy. For this purpose, we compared results obtained with the proposed algorithm for different selections from the measured data set for the porcine Achilles tendon (see Figure 5). The resulting histograms in the lower left corner of the micrographs are fitted with von Mises distribution function for which mean direction *μ*, concentration parameter *b*, and coefficient of determination *R*^2^ are listed in the figures. The percentage indicates the portion of evaluated pixels; as Achilles tendons consist dominantly of collagen fibers, all pixels should be evaluated except for holes between the individual fiber bundles or tissues other than collagen.

Although in all the compared variants the mean angle is evaluated correctly, there are significant differences in the other parameters of the distribution. While the first three micrographs are almost identical in all parameters, the last histogram (based on 3 images only) suffers from a lower percentage of evaluated pixels, a significant number of erroneously evaluated angles (rotated by 90°), and consequently a different concentration parameter *b*. For the histogram based on 4 images, these errors are also significant although lower. In contrast, 5 images seem to give correct parameters of the distribution but a higher percentage of not evaluated pixels (i.e., the pixels which fell out because their Pearson coefficient was below a set minimum threshold and the fiber orientation could not be determined reliably) indicates a risk of errors when evaluating less homogeneous tissues. Consequently, 6 images (corresponding to 30° rotation step) were decided to represent the necessary minimum to be recorded with both perpendicular and rotated polarizers. This may seem to contradict our earlier statement that 3 images are sufficient for the correct evaluation of minima within the *π*/2-periodicity of light intensity profile recorded with perpendicular polarizers [28]. However, this inconsistency is due to our decision to keep the number of images in both sets (with perpendicular and rotated polarizers) the same in order to avoid operator errors and increase the accuracy. Although we did not explore it, the variant with 3 images in the first and 6 images in the second set could further reduce the total number of images required to determine the fiber orientation with adequate accuracy.

**Figure 6:**
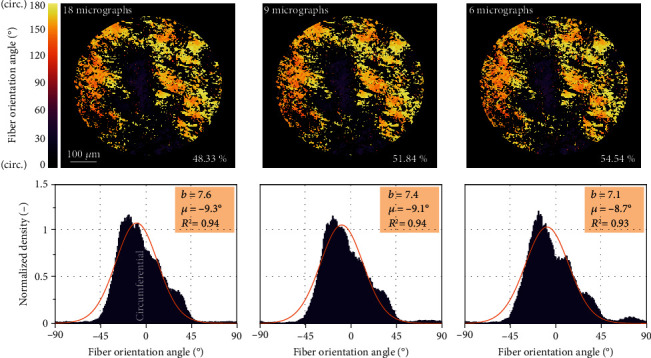
Comparison of the resulting histograms of collagen azimuthal angles in porcine aorta obtained with different numbers of input micrographs.

Finally, to check the suitability of the algorithm for tissues with a more complex collagen arrangement, we performed the same comparison for the media of porcine aortic wall. The evaluated sections are in the circumferential-axial plane with dispersion and waviness being much higher than in radial direction and thus more difficult for evaluation [13, 41]. Here, only some half of the pixels was evaluated, matching the percentage of collagen in this layer [42]. The histograms in Figure 6 confirm that the mean angle and concentration parameter of the distribution were evaluated correctly and accurately in all cases. We can conclude that 6 rotated micrographs are sufficient for the evaluation of comparable tissues to avoid the reduction in the number of evaluated pixels or incorrect evaluation of their directions.

## 4. Discussion

In this paper, we have proposed a new semiautomated method for evaluation of the orientation and dispersion of collagen fibers in soft tissues. It requires ca two minutes to record manually the needed 12 pictures of one micrograph and then it takes a few seconds to evaluate the orientation in up to 5·10^5^ pixels. The presented method overcomes two major limitations of PLM: time-consuming manual evaluation and the *π*/2-periodicity of light intensity with crossed polarizers. The used optical equipment—polarized light microscope with digital camera and 2D rotary stage—allowed us to evaluate reliably the orientation of collagen fibers in each pixel of the micrograph. For comparison, the study [43] evaluated less than 100 points (areas) per histogram; in contrast, [27] used a manual method using PLM and evaluated a total of 5040 collagen orientations (30 different points at each of these slices).

To prove the correctness of the algorithm, it was verified using several manually evaluated pixels in micrographs of porcine Achilles tendon with mostly unidirectional and straight fibers. The subsequent validation against manual measurements with the same specimens demonstrated benefits and efficiency of the proposed technique. In the manual evaluation, we could not evaluate more than some 300 areas per microscopic image, while the algorithm determined the fiber orientation in each pixel, i.e., some 4.5·10^5^ values in the same image. The much higher *R*^2^ values of von Mises distribution functions used for histogram approximations confirm a significantly better quality of structural parameters fitted to such histograms in comparison with the manual measurements. The robustness of the algorithm was highlighted also when the reduction of the needed number of rotated images was tested. The differences in structural parameters obtained on the basis of different sets of images were negligible (see Figure 5) when at least 6 rotated images were used, thus, establishing this number as optimum. This was the case not only with the highly uniformly oriented tendon tissue but also for the aortic wall with much more dispersed and curved collagen fibers. Although these histograms showed a visible asymmetry, their fitting with (symmetric) von Mises distribution function gave coefficient of determination *R*^2^ > 0.93 in all cases, and the resulting structural parameters were independent of the chosen number of images. Thus, the presented automated algorithm enables us to gain large data sets describing the in-plane distribution of collagen fibers in soft tissues and consequently also the reliable structural parameters needed for the structure-based constitutive description of collagenous tissue in computational modeling. The well-founded structural parameters significantly improve the quality of computational models of soft tissues, which in turn play an extremely important role in simulations of arteries under pathological conditions. As an example, these models facilitate the evaluation of rupture risk of aortic aneurysm [4, 23] or fibrous cap of carotid atherosclerotic plaque [44] and may help in planning of surgeries.

In contrast to QPLM [20, 21], CLSM [13, 14], or MM [15], the proposed method does not require any additional equipment such as quarter-wave plates [34, 35] and compensators [29–33] which may be difficult or even impossible to be supplemented into some of the microscopes as it was in our case; it is based on light microscopy and requires only two polarizers and a standard rotary table. Thus, the proposed method appears promising thanks to its broad applicability even with the simplest polarized light microscopes.

Some limitations remain, of course, mostly due to the basic principles of light microscopy. The thickness of specimens must be lower than 10 *μ*m, thus, their cutting with microtome may damage some structural components or tear fibers, especially if their out-of-plane dispersion or waviness are significant. The applied 5 *μ*m thin slices are also very compliant, and their position on the glass substrate may be distorted and thus vary locally. In comparison with these variations, the inaccuracies caused by manual settings of the angles of polarizers and specimens are much lower (<1°). Also, we neglect the strains caused by unfolding of the rounded aortic specimens. Other errors could be introduced through misalignment of the polarizers or inaccurate positioning of the sample. A careful manipulation, however, may reduce these errors to a level insignificant in comparison with fiber dispersion and tissue variability. Despite these sources of errors, our procedure evaluated correctly the orientation of fibers, proving its robustness and effectivity. Naturally, errors related to extreme nonhomogeneity of the tissue, such as reported by Jett et al. [45], cannot be eliminated and must be treated by subdivision of the specimens.

The objective of the proposed method, similarly to most of the others, is to obtain histograms of fiber directions. For their following transformation into structural parameters of the constitutive models, we have shown here only approximation with unimodal von Mises distribution function. Naturally, for 3D distributions or for multimodal distributions (with more fiber families), more complex distribution functions have to be applied. However, it is not quite easy to distinguish between fiber waviness and dispersion, and misinterpretations of wavy fibers as two fiber families may occur [28]. Thus, completely different approaches should be adopted to obtain also parameters representing fiber waviness [15] that appear in some constitutive models (for instance [46]). These approaches may be based on histological investigation of the tissue under load, when the fibers are more or less straightened [5, 6, 44, 45]; this issue is, however, out of scope of this paper. Transformation of histograms into structural parameters of constitutive models is addressed in greater detail in another paper being just prepared.

In the context of our experiments, a higher resolution of the camera was not needed, since we do not study collagen fibers at the molecular level, and the large view-field allowed us to quickly evaluate the examined area of the sample. Nevertheless, it is possible to easily enlarge the studied area using special objectives or a larger camera chip. Conversely, for applications in which high resolution is important, one can choose an objective with a high numerical aperture and magnification.

## 5. Conclusion

We demonstrated a new method for semiautomatic, fast, and accurate evaluation of orientation and dispersion of collagen fibers in soft tissues using polarized light microscopy. Our method is based on two sets of six rotated micrographs obtained with both perpendicular and rotated polarizers. The mutual rotation of both polarizers overcomes the *π*/2 ambiguity in the fiber orientation inherent to their orthogonal configuration and given by the *π*/2-periodic intensity function of the polarized light. The prospect of this technique is to enable all the labs with standard equipment a fast evaluation of collagen orientation in the tissues without need of any additional components. In human biomechanics, it will facilitate the evaluation of the structural constitutive parameters of blood vessel walls and other collagenous tissues which can be further used in computational modeling of their mechanical behavior under healthy and pathological conditions.

## Data Availability

Data underlying the results presented in this paper are not publicly available at this time but may be obtained from the authors upon reasonable request. Also, the algorithm for fiber direction evaluation can be provided by the authors on request.

## Supplementary Materials

Supplementary Materials The manuscript includes additional material describing the mathematical nature of polarized light microscopy.
