# Supplementary material for: Full-Range Optical Imaging of Planar Collagen Fiber Orientation Using Polarized Light Microscopy
Source: Biomed Res Int. 2021 Nov 28;2021:6879765. doi: 10.1155/2021/6879765 (PMC8645375; doi:10.1155/2021/6879765)
Supplement: Supplementary Materials — The manuscript includes additional material describing the mathematical nature of polarized light microscopy. [file 6879765.f1.docx]

Full-range optical imaging of planar collagen fiber orientation using polarized light microscopy: supplementary material

**Mathematical nature of polarized light** **microscopy**

The mathematical-physical basis of PLM is presented here in greater detail. The unpolarized light passes through the first polarizer P1 oriented along the x-axis, then through a sample characterized by a transmission matrix *α* (generally complex) and finally through the second polarizer P2 (see Fig. 1a). The rotation of the sample coordinate system with respect to the global one is specified by angle *θ*. The collagen fiber is implicitly assumed to coincide with the horizontal axis of the sample coordinate system (and with the orientation of the first polarizer), although generally, its orientation with respect to the sample coordinate system (in the main text denoted as *p*) is arbitrary and it is, in fact, the very parameter we strive to determine. Here, we set its value to zero to keep the notation simple and lucid, knowing it only leads to a shift in the angular intensity profile without any effect on its shape. The default orientation of the second polarizer is along the y-axis (the cross-polarized configuration of the microscope) and the deviation of P2 from this default orientation is denoted as angle *δ*. The electric field vector ***E*** emerging from the setup can be calculated using the following matrix formalism:

|  | $\boldsymbol{E}=R^{-1}\left( \delta\right)P_{2}R\left( \delta\right)R^{-1}\left( \theta\right)\alpha R\left( \theta\right)\boldsymbol{E}_{0}$, | (S1) |
| --- | --- | --- |

where the individual matrices have the meaning as follows:

Incident wave (after passing the first polarizer):

|  | $\boldsymbol{E}_{0}=\binom{1}{0}$, | (S2) |
| --- | --- | --- |

rotation matrix of the sample:

|  | $R\left( \theta\right)=\left( \begin{matrix} \cos\theta& \sin\theta\\ -\sin\theta& \cos\theta\end{matrix} \right),$ | (S3) |
| --- | --- | --- |

rotation matrix of the P2:

|  | $R\left( \delta\right)=\left( \begin{matrix} \cos\delta& \sin\delta\\ -\sin\delta& \cos\delta\end{matrix} \right),$ | (S4) |
| --- | --- | --- |

and for $\delta=0^{\circ}$ rotation matrix of the P2:

|  | $R\left( \delta=0^{\circ} \right)=\left( \begin{matrix} 1 & 0 \\ 0 & 1 \end{matrix} \right).$ | (S5) |
| --- | --- | --- |

Transmission matrix:

|  | $\alpha=\left( \begin{matrix} \alpha_{xx} & \alpha_{xy} \\ \alpha_{xy} & \alpha_{yy} \end{matrix} \right),$ | (S6) |
| --- | --- | --- |

$P_{2}$ matrix:

|  | $P_{2}=\left( \begin{matrix} 0 & 0 \\ 0 & 1 \end{matrix} \right)$. | (S7) |
| --- | --- | --- |

For the cross-polarized configuration ($\delta=0$), the electric field attains the following simple form

|  | $\boldsymbol{E}=\binom{0}{\frac{1}{2}\left( \alpha_{xx}-\alpha_{yy} \right)\sin2\theta+\alpha_{xy}\cos2\theta}.$ | (S8) |
| --- | --- | --- |

In the experiment, the signal practically vanishes for values of *θ* equal to a multiple of $\frac{\pi}{2}$, therefore we will further neglect the off-diagonal terms of transmission matrix *α* (this means that the sample does not change the polarization state of the passing light when the collagen fibers are parallel or perpendicular to the polarization of the incident field). Consequently, the transmission matrix assumes a form coincident with a simple birefringent material.

The signal at the camera/detector is proportional to the intensity of the light emerging from the setup. Assuming the simplified expression for the transmission matrix, we obtain

|  | $I=\left\vert\boldsymbol{E} \right\vert^{2}=\frac{1}{8}\left\vert\alpha_{xx}+\alpha_{yy} \right\vert^{2}\left( 1-\cos2\delta\right)+\frac{1}{8}\left\vert\alpha_{xx}-\alpha_{yy} \right\vert^{2}\left( 1-\cos4\theta\cos2\delta-\sin4\theta\sin2\delta\right)+\frac{1}{4}\left( \left\vert\alpha_{xx} \right\vert^{2}-\left\vert\alpha_{yy} \right\vert^{2} \right)\left[ \cos2\theta\left( 1-\cos2\delta\right)-\sin2\theta\sin2\delta\right].$ | (S9) |
| --- | --- | --- |

Although we use this expression in our calculations, it is insightful to take a limit for small values of *δ* (by expanding the trigonometric function into Taylor series and retaining only the lowest terms). Then the approximate expression for the light intensity reads

|  | $I\left( \theta\right)\approx\frac{1}{8}\left\vert\alpha_{xx}-\alpha_{yy} \right\vert^{2}\left[ 1-\cos\left( 4\left( \theta-p \right) \right) \right]-\frac{1}{2}\delta\left( \left\vert\alpha_{xx} \right\vert^{2}-\left\vert\alpha_{yy} \right\vert^{2} \right)\sin\left( 2\left( \theta-p \right) \right).$ | (S10) |
| --- | --- | --- |

The first term represents the basic profile obtained for the cross-polarized configuration ($\delta=0$). Its periodicity is $\frac{\pi}{2}$ and one therefore cannot distinguish between the 45° and 135° orientations of the collagen fibers. Also note that it vanishes for $\alpha_{xx}=\alpha_{yy}$ which illustrates the necessity to use birefringent materials in these experiments. The second term has a periodicity of π and for samples with $\left| \alpha_{xx} \right|\neq\left| \alpha_{yy} \right|$ it can account for the different spectra of colors observed for two perpendicular collagen fiber orientations.

By inspecting Eq. (S9) we can see that for one of the above orientations of collagen fibers (45°) the intensity of green color will increase while we should observe a decrease for the other one (135°), which nicely corresponds to the behavior observed in the experiment.

To verify this hypothesis, we used the full expression for the light intensity given by Eq. (S9) and compared our analytical model with measurements performed for different values of *δ* (see Fig. 2). Transmission intensities $\left| \alpha_{xx} \right|^{2}$ and $\left| \alpha_{yy} \right|^{2}$can be measured directly using the configuration with aligned polarizers. The term $\left| \alpha_{xx}-\alpha_{yy} \right|^{2}$ can be obtained by fitting the measurements for $\delta=0^{\circ}$. The last unknown quantity $\left| \alpha_{xx}+\alpha_{yy} \right|^{2}$ is then easily obtained from the equality

|  | $\left\vert\alpha_{xx}+\alpha_{yy} \right\vert^{2}+\left\vert\alpha_{xx}-\alpha_{yy} \right\vert^{2}=2\left( \left\vert\alpha_{xx} \right\vert^{2}+\left\vert\alpha_{yy} \right\vert^{2} \right)$. | (S11) |
| --- | --- | --- |

As for the particular angular profiles presented in Fig. 2, the transmission intensities $\left| \alpha_{xx} \right|^{2}$ and $\left| \alpha_{yy} \right|^{2}$ were found to be 1645 and 3403 counts at the 550 nm wavelength, respectively. The $\delta=0^{\circ}$ measurement yielded an estimated value of 2096 counts for the term $\left| \alpha_{xx}-\alpha_{yy} \right|^{2}$ (calculated as the maximum peak value multiplied by 4). The use of the above equality then sets the value of $\left| \alpha_{xx}+\alpha_{yy} \right|^{2}$ to 8000 counts. It should be stressed that for this calibration procedure to be valid, all the measurements need to be performed with the same camera settings.
